# A chemically fuelled self-replicator

**DOI:** 10.1038/s41467-019-08885-9

**Published:** 2019-03-01

**Authors:** Sarah M. Morrow, Ignacio Colomer, Stephen P. Fletcher

**Affiliations:** Department of Chemistry, Chemistry Research Laboratory, University of Oxford, Mansfield Road, Oxford, OX1 3TA UK

## Abstract

The continuous consumption of chemical energy powers biological systems so that they can operate functional supramolecular structures. A goal of modern science is to understand how simple chemical mixtures may transition from non-living components to truly emergent systems and the production of new lifelike materials and machines. In this work a replicator can be maintained out-of-equilibrium by the continuous consumption of chemical energy. The system is driven by the autocatalytic formation of a metastable surfactant whose breakdown products are converted back into building blocks by a chemical fuel. The consumption of fuel allows the high-energy replicators to persist at a steady state, much like a simple metabolic cycle. Thermodynamically-driven reactions effect a unidirectional substrate flux as the system tries to regain equilibrium. The metastable replicator persists at a higher concentration than achieved even transiently in a closed system, and its concentration is responsive to the rate of fuel supply.

## Abstract (web summary)

Understanding how simple chemical mixtures transition into truly emergent systems is essential to create new lifelike materials. Here, the authors show a self-replicating system that can be maintained out-of-equilibrium by an oxidant fuel in analogy to simple metabolic cycles.

## Introduction

The structures and functions of even the simplest living organisms seemingly operate counter to the maximisation of entropy and rely on the consumption of chemical energy to maintain dissipative states^1–3^. The behaviour of out-of-equilibrium biological systems has inspired many synthetic supramolecular materials and networks^4–7^ and enabled function such as self-healing and adaptation in materials^8^, controlled directional motion^9–12^, catalysis^13^, primitive selection^14^, and oscillatory behaviour^15–17^. However, although self-replication^18,19^ under out-of-equilibrium regimes is indispensable to life, it is not well developed in synthetic systems^20,21^.

**Fig. 1:**
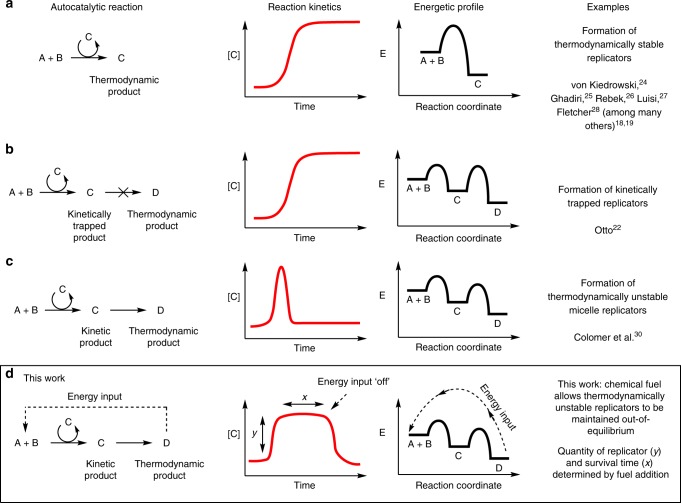
The dynamics of some known self-replicators. **a** Autocatalyst **C**, or self-replicator **C**, is a catalyst for its own formation and is thermodynamically stable. **b** Self-replicator **C** promotes its own formation from a dynamic library of building blocks and products via supramolecular interactions, forming a kinetically trapped set of replicators^22^. **c** Self-replicator **C** is a catalyst for its own formation and is the kinetic product of the reaction. It is eventually destroyed to give thermodynamic, non-replicating, product **D**^30^. **d** This work: A replicator **C** that is both formed and destroyed can be maintained, out-of-equilibrium, by a chemical fuel. The quantity of replicator (*y*) and survival time (*x*) are determined by the addition of fuel

Many synthetic small molecule template and supramolecular replicators have been developed with analogy to biological systems^18,19^, demonstrating emergent traits such as heritability^22^ and selection^23^. However, these replicators still invariably form thermodynamic (Fig. 1a) or kinetically trapped products (Fig. 1b)^24–28^.

Stable replicators (Fig. 1a), which are not subject to a destruction reaction, will move to equilibrium after the consumption of starting materials. Whilst kinetically trapped replicators are not at the thermodynamic minimum, they are also not in an energy-consuming, dissipative, out-of-equilibrium state (Fig. 1b). For example, macrocyclic disulfides within a dynamic combinatorial library, as developed by Otto, are formed autocatalytically via fibrous stacks, whose breakage and exposure of fibre ends leads to an increase in the macrocyclic building blocks (Fig. 1b). The final distribution of macrocycle replicators is static and explicitly shown to be a kinetically trapped state under the conditions of growth^22,29^. No source of energy is required to maintain this distribution and it is not a dissipative out-of-equilibrium state.

Metastable replicators, which are subject to the continuous pressure of degradation (Fig. 1c, d)^30^, provide the opportunity to devise and study out-of-equilibrium states, as long as strategies for keeping these systems out-of-equilibrium can be realized. However, devising systems featuring the required formative and destructive steps, and which consume an appropriate fuel to drive out-of-equilibrium assembly, is not trivial^4–7^. A large number of dissipative supramolecular systems operate under the control of light and many are limited by the batch supply of fuel. Systems operating under a continuous consumption of a chemical fuel would have greater similarity to biological dissipative structures^7^.

The design of replicators which are subject to a chemical destructive step arrived in 2001 with work by Luisi et al. on vesicles^31–33^ and in our recent work on the formation of replicating micelles by alkene metathesis^30^. These systems involve the self-organisation of surfactant molecules into aggregates capable of catalysis, which has long been considered important for primitive life forms^18,27,28,34–37^.

Both reports relied on the incorporation of a kinetically accessible chemical destruction step which destroyed the system’s ability to replicate (Fig. 1c). However, in neither report was the replicator maintained out-of-equilibrium. Two additional requirements were lacking; first, the high-energy replicator must be regenerated by the input of fuel. Trivially, this might be achieved by the external addition of more starting materials, but a more advanced system could regenerate precursors internally in a form of metabolism. Second, once this fuel supply is switched off, the out-of-equilibrium species must be degraded, demonstrating that it is the fuel that maintains the non-equilibrium state.

**Fig. 2:**
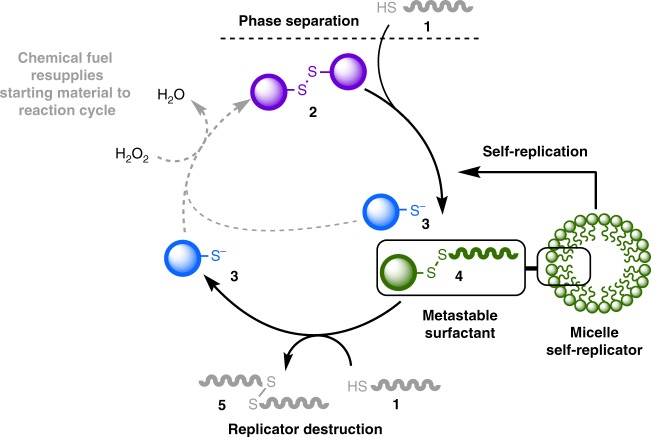
The substrate cycle of a chemically fuelled self-replicator. Reaction between phase-separated components **1** and **2** is slow and forms thermodynamic waste product **3** and surfactant **4**. Aggregation of **4** into micelles increases phase mixing and the rate of reaction to form additional **4** so that surfactant formation is autocatalytic and the supramolecular aggregates self-replicating. Destruction of **4** by a second thiol-disulfide exchange forms additional waste **3** and **5**. At equilibrium, in a closed system (solid arrows), the surfactant **4** is destroyed. However addition of an oxidising fuel (dashed grey arrows) regenerates starting disulfide **2** from waste **3** and allows the replicator population to be maintained and function as a replicator in an out-of-equilibrium state. At the steady state successive cycles consume both oxidising fuel and thiol **1** whilst intermediates **2**, **3** and **4** remain at the same concentration—this is similar to substrate cycles observed in biological systems^39,40^. Once fuel supply is halted the replicator stops being re-formed and the system degrades to equilibrium

In this work, we demonstrate the maintenance of a self-replicator out-of-equilibrium in a dissipative self-assembled state (Figs. 1d and 2). Our system design does not require externally added reagents for the destructive step, nor an external addition of starting materials. Instead, a single, simple chemical fuel (an oxidising agent) is used to regenerate high-energy starting materials in situ from waste byproducts of the reaction, establishing a simple metabolic cycle (Fig. 2) to maintain the replicator out-of-equilibrium. Halting the fuel supply results in the complete degradation of the surfactant replicator; it is the simple fuel supply that enables the self-replicator to persist in time and to retain or regain replicator function, away from equilibrium. Higher concentrations of the replicator can be achieved in the fuel-consuming out-of-equilibrium regime than can be even transiently observed in a closed system, and the concentration of the replicator at the steady state is adaptable to variations in the fuel supply.

Our self-replicating system relies on the reaction between phase-separated components to form a surfactant^28,30,37,38^. Reaction between phase-separated hydrophobic octanethiol **1** and hydrophilic disulfide **2** is slow but produces mixed disulfide surfactant 2-nitro-5-(octyldisulfaneyl)benzoic acid **4** (Fig. 2). Once a certain concentration of surfactant **4** has been reached micellar aggregates form and increase mixing between the phases, increasing the rate of reaction to form their own components. In this way the aggregates act as catalysts for their own formation and self-replicate. This spontaneous reaction causes the anabolic construction of **4** and micelles of surfactant **4**. Formation of **4** also produces thermodynamically stable by-product **3**.

Key to the design of our out-of-equilibrium system is that the replicator undergoes a second destructive, or catabolic, step to breakdown surfactant **4**. This second spontaneous, but slower, thiol-disulfide exchange with **1** expels a second equivalent of thermodynamic product **3** and forms a non-functional waste product **5** (Fig. 2). Without the regeneration of replicator precursor **2**, the equilibrium position of the system is therefore for complete destruction of the replicator to give **3** and **5**.

However, thermodynamic products **3** can act as precursors for regenerating the complex supramolecular system when supplied with an oxidant. A second anabolic step rebuilds high-energy starting material **2** upon addition of oxidising fuel, and the replicating system can be maintained out-of-equilibrium (Fig. 2). Octanethiol **1** is not regenerated during the cycle but is present in excess from the beginning of reaction to act both in the replicator formation and destruction steps so that resupply of reagent **1** is not required. The fuel regenerates the limiting precursor for the replicator so as to establish a metabolic cycle, similar to substrate cycles seen in biological systems^39,40^. This continuous cycle of fuel consumption and waste production is the key to maintaining the high-energy, self-assembling, self-replicator in an out-of-equilibrium state.

## Results

### Surfactant self-replication

**Fig. 3:**
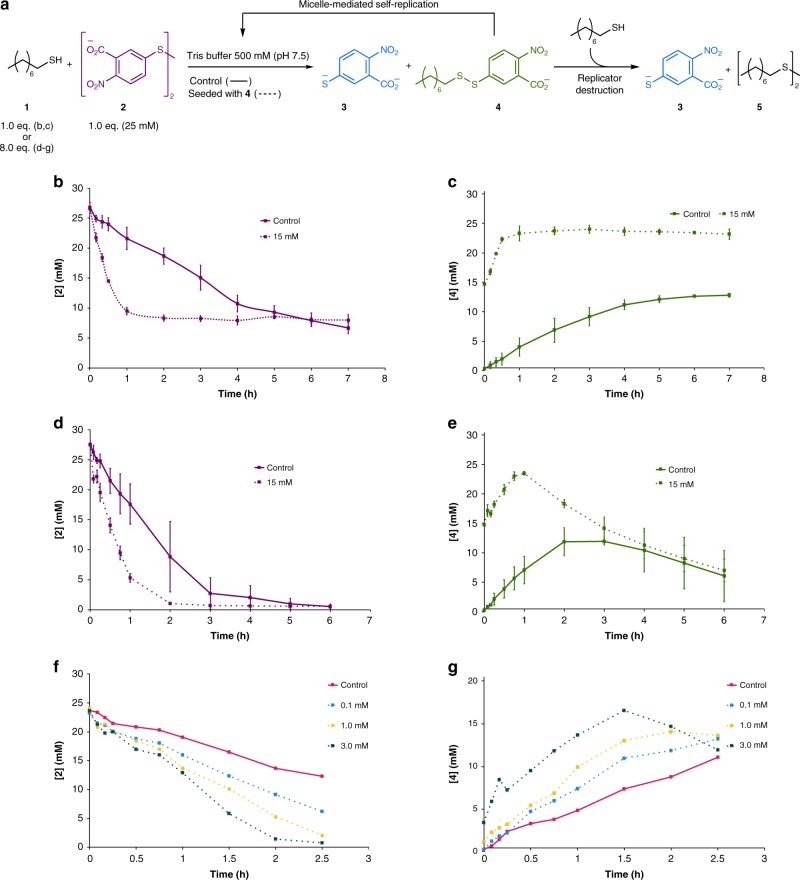
Control and seeded reactions. **a** A series of control and seeded reactions were performed to demonstrate autocatalysis, with variation both in the concentration of seeded product **4** and the equivalents of octanethiol **1**. **b**, **c** Concentration of **2** and **4** (respectively) over the course of the reaction conducted with 1.0 equivalent of octanethiol **1**, under control (solid line) and seeded (15 mM **4**, dotted line) conditions. **d**, **e** Concentration of **2** and **4** (respectively) over the course of the reaction conducted with 8.0 equivalent of octanethiol **1**, under control (solid line) and seeded (15 mM **4**, dotted line) conditions. **f**, **g** Concentration of **2** and **4** (respectively) over the course of the reaction under control (solid line) and seeded (various concentrations **4**, dotted lines) conditions. The concentration of reaction components was monitored by UPLC. Each plotted point in **b**–**e** is the average of three reactions and the error bars represent the standard deviation. For **f** and **g** reactions were performed once only

The ability of micelles of **4** to self-replicate was demonstrated by comparing the rates of starting material consumption and product formation between control reactions containing only **1** and **2** and reactions containing **1**, **2** and seeded **4** (Fig. 3).

Reactions were first conducted with a relatively high concentration of seeded **4** (15 mM). Two reaction conditions were tested: 1.0 and 8.0 equivalents of octanethiol **1** (Fig. 3). Under both conditions the formation of new **4** and the consumption of **2** were observed to be faster than the control reaction (compare dotted and solid lines in Fig. 3b–e); the effect was most evident where only 1.0 equivalent of octanethiol **1** was used (c.f. Figure 3b–e). Extracted initial rates are shown in Supplementary Figures 8 and 9.

Further reactions were conducted using 8.0 equivalents of octanethiol **1** at lower concentrations of seeded **4**, from 0.1 to 3.0 mM, testing the catalytic power of the surfactant (Fig. 3f, g). Under these conditions rate of the consumption of **2** and the production of **4** increased as the concentration of seeded **4** increased.

In this system the relationship between the reaction rate and the quantity of seeded product is not simple. Most importantly, the presence of the second degradation step, which will vary in influence with the concentration of seeded **4** as well as the concentration of **1**, affects the observed rate of both the consumption of **2** and production of **4**. Unfortunately, this makes the extraction and comparison of initial reaction rates with different quantities of seeded **4** not very meaningful. What is clear, however, is that seeding the reaction with **4** from the beginning of the reaction observably increases the reaction rate and the production of new **4** (see also Supplementary Figure 10).

With analogy to our previous studies^28,30,37,38^, inspired by the work of Luisi and others^27,41^, the acceleration is ascribed to the formation of micelles of **4** which increase mixing between the phase-separated reactants. The formation of micelles from pure, isolated **4** (measured in 500 mM Tris buffer at pH 7.5) was confirmed by dynamic light scattering (DLS), with the observation of aggregates of hydrodynamic diameter between 15 and 20 nm depending on concentration (Supplementary Figures 1–3). Under these idealized conditions the critical micelle concentration (cmc) of **4** was about 0.2 mM as determined by ring tensiometry (Supplementary Figure 4). The mechanism of these aggregate-catalysed biphasic reactions is complex, and the reactions are sensitive. The aggregate catalysts are dynamic with the continuous movement of surfactant between aggregate and free solution, and the precise location of the catalysed reaction, whether within the micelle or in free solution, is difficult to determine^18^. Detailed study of these reactions at the single aggregate level has been undertaken by our group to refine our understanding of these systems^37^. Nevertheless, the emergent behaviour of the system as a whole is easy to visualise, as shown in Fig. 3.

### Closed system behaviour

**Fig. 4:**
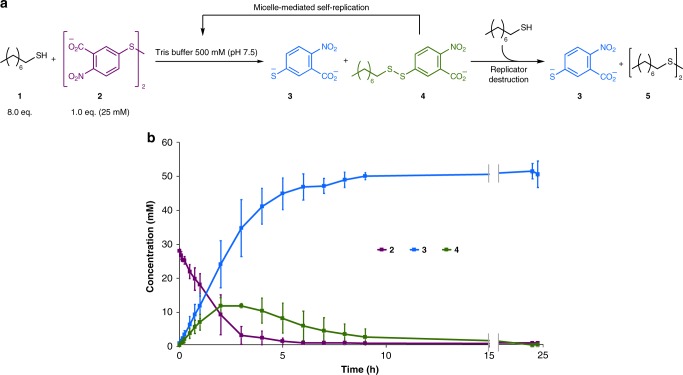
Closed system behaviour: the destruction of the replicator. **a** Thiol-disulfide exchange between neat octanethiol **1** and aqueous disulfide **2** produces a mixed disulfide surfactant **4** and anionic by-product **3**. Micelles of surfactant **4** increase mixing between the phase-separated reactants and catalyse the reaction to form their own components. However, **4** is subject to a destruction reaction to form thermodynamic products **3** and **5**. **b** Concentration of all UV active species **2**, **3**, and **4** over the full course of reaction. Surfactant **4** is unstable towards attack by additional **1** to give waste products **3** and **5** and the equilibrium position of the system is for complete degradation of **4**. The concentration of reaction components was monitored by UPLC. Each plotted point is the average of three reactions and the error bars represent the standard deviation

The closed system behaviour of the reaction and the concentration of all reaction components is shown in Fig. 4. At the beginning of the reaction the formation of **4** is observed, and **4** reaches a maximum concentration before its destruction to **3** and **5** in the presence of excess **1**. The thermodynamic drive to **3** and **5** is primarily due to the electron withdrawing groups on thiolate **3** (which stabilize this anion, making **3** a good leaving group and poor nucleophile)^42^. The thermodynamic driving force of these reactions makes them effectively irreversible; this irreversibility provides directionality for the metabolic replicator cycle and the system as a whole. The clockwise flux of substrates in the cycle (Fig. 2) reflects the system’s desire to regain the equilibrium position, moving in the direction of irreversible reactions to thermodynamic products.

In contrast to the surfactant formation step, the second, slower, thiol-disulfide exchange does not appear to be accelerated by micelle formation. This was found by monitoring the rate of a series of control reactions between octanethiol **1** and surfactant **4** at different concentrations of **4** both below and above the cmc (Supplementary Figures 11–13).

### Self-replication out-of-equilibrium

Closure of the substrate cycle in Fig. 2 is achieved by regenerating starting disulfide **2** in situ by oxidation of waste **3** with slowly added 1.2 M hydrogen peroxide. The phase separation allows supply of the fuel to the aqueous phase (by addition with a syringe pump) to selectively oxidize **3** while avoiding oxidation of **1**, which would likely otherwise form non-functional **5**. The phase separation is therefore important for the observation of self-replication and the establishment of the chemically fuelled substrate cycle.

**Fig. 5:**
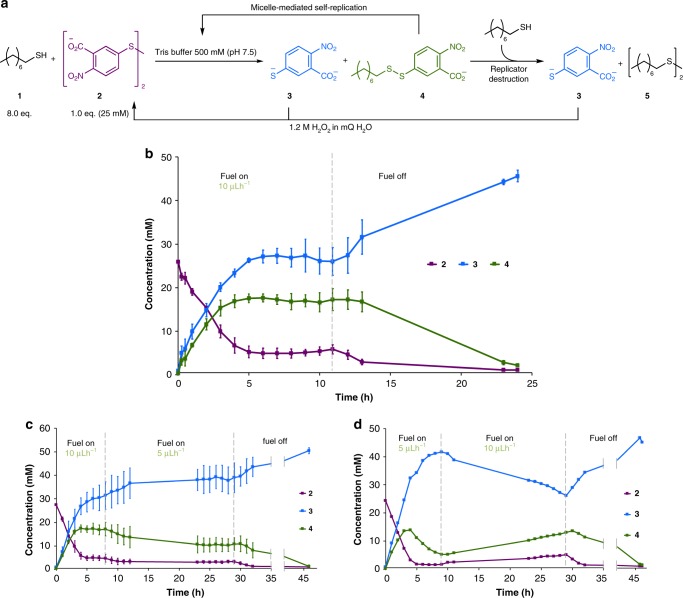
Self-replication maintained out-of-equilibrium by addition of a chemical fuel: **a** Chemical structures and reactions involved in the out-of-equilibrium system. **b** Continuous supply of an oxidising fuel to the aqueous phase (by syringe pump) completes the substrate cycle and allows **4** to reach a steady state. By the chemically fuelled continuous regeneration of **2** the rates of formation and destruction of surfactant **4** can be balanced so that while individual molecules are continuously formed and destroyed the overall concentration remains steady. Once fuel supply is halted, the entire system degrades towards equilibrium with the destruction of **4** by excess **1** and the formation of waste products **3** and **5**. **c** Initial rate of oxidant supplied was 10 μL h^−1^ for 8 h, then 5 μL h^−1^ for 21 h. Lowering the rate of fuel supply causes the system to adapt to a new steady state with a lower concentration. Less fuel effectively limits the starting materials for the autocatalytic reactions; less **2** is available and **4** can no longer maintain as high a concentration. Once the fuel supply is halted, the entire system degrades towards equilibrium. **d** After an initial rate of fuel supply of 5 μL h^−1^ for 9 h, the rate was raised to 10 μL h^−1^ for a further 20 h. Increasing fuel supply allows the system to support higher replicator concentrations and maintain a position further removed from equilibrium. The concentration of reaction components was monitored by UPLC. Each plotted point in **b**, **c** is the average of three reactions and in **d** of two reactions. Error bars represent the standard deviation

By continuously providing fuel it is possible to establish a steady population of **4** as well as control the population of **4** (Fig. 5). Remarkably, a higher concentration of **4** can be maintained under chemically fuelled conditions than is observed (even transiently) under closed system conditions where the starting materials are simply allowed to react (c.f. Figures 4b and 5b). Under fuelled conditions, building block **2** is never completely depleted as a consequence of recycling **3**. Formation of **4** from **1** and **2** continues to compete with destruction, resulting in a balance in the rates of formation and destruction of **4**, whose concentration as a whole remains stable and elevated. The elevated concentration of **4** relative to the transient peak in the closed system can be explained by the fact that its formation rate is proportional to the concentration of **2**. Under fuelled conditions, oxidation of **3** decreases [**3**], increasing [**2**], and therefore the rate of formation of **4**. The destruction of **4** is inherently slower (as demonstrated by the fact that **4** is formed at any significant concentration), and so **4** reaches a higher concentration before the destruction step can catch up at the steady state.

Upon stopping the fuel supply the entire system decays toward equilibrium so that **4** is completely consumed, demonstrating that the observed steady state concentrations of **2**, **3**, and **4** are not equilibrium nor kinetically trapped positions. Chemical fuel energy consumed by the system is therefore used to drive the metabolic cycle, via the production of components that are thermodynamically and/or kinetically unstable; halting the fuel supply stops regenerating **2** and results in movement towards the equilibrium state.

The current system does not regenerate octanethiol **1** and one equivalent is consumed in each course of the substrate cycle. The system therefore requires an excess of nucleophile **1**, which also, importantly, provides the necessary phase separation, although as further reaction cycles are completed phase separation should be maintained by generation of **5**. We point out that if **1** were completely consumed then the reaction mixture, after halting of fuel supply and final equilibration, would be expected to contain some **4**.

Varying the fuel supply can be used to control the concentration of **4**. When fuel is supplied at 5 μL h^−1^ (instead of 10 μL h^−1^) the system is no longer able to support as high a concentration of **4** – fewer building blocks are produced in the substrate cycle – so **4** adopts a lower concentration. Here, halting the fuel supply again causes the replicator to die out and the system degrades to equilibrium (Fig. 5c). Similarly, the system is able to recover from an initially limited supply of fuel to achieve higher concentrations of **4** when fuel is more abundant (Fig. 5d). At an initial rate of 5 μL h^−1^ the replicator is little able to counteract the degradation towards equilibrium and only a low concentration of **4** is supported. However, increasing the fuel supply to 10 μL h^−1^ allows **4** to recover. Again, finally halting the fuel supply allows the system to move to equilibrium.

In conclusion, we have established a system of self-replicating micelles that can be maintained in an out-of-equilibrium state by the action of a chemical fuel. The production of a functional aggregate system capable of catalysis and replication has long been thought of as important for the origins of life, but for more lifelike dynamics such systems must exist in dissipative states. This work establishes the conditions required for metastable replicators to persist by the continuous consumption of chemical energy. Here, a chemical fuel is used to establish a metabolic substrate cycle, reminiscent of simple metabolic pathways observed throughout biology. Fuel consumption shifts the replicator into an out-of-equilibrium state by the continuous regeneration of building blocks for further replicator formation. Energy consumption maintains the replicator concentration, which is adaptable to variations in fuel supply and can even be elevated above the highest concentration observed in a non-fuelled or closed system. The persistence and adaptability of the self-replicator has obvious analogies with the maintenance of high-energy biological structures, such as microscale microtubules or a macroscale self-replicating organism.

## Methods

### General experimental details

Reagents **1** and **2** and Trizma^®^ base obtained from Sigma-Aldrich were used directly as supplied (unless indicated otherwise as detailed in procedures). All anhydrous reactions were carried out in flame-dried glassware and under an inert atmosphere of argon provided by a balloon or Schlenk line. All reactions were stirred with magnetic followers. CH_2_Cl_2_, when used as a reaction solvent, was dried by purification through two activated alumina purification columns. Flash column chromatography was performed using silica gel (60 Å, 0.033–0.070 mm, BDH). TLC analyses were performed on Merck Kiesegel 60 F_254_ 0.25 mm precoated silica plates.

### Compound synthesis and characterisation

^1^H NMR and ^13^C NMR spectra were recorded on a 400 MHz spectrometer at room temperature in (CD_3_)_2_SO, D_2_O and CDCl_3_ and referenced to residual solvent peaks. Infrared spectra were recorded as thin films of neat samples on a Bruker Tensor 27 FT-IR spectrometer equipped with Attenuated Total Reflectance sampling accessories. High-resolution mass spectra were recorded on a Bruker MicroTof (resolution = 10,000 FWHM) under conditions of electrospray ionization (ESI) or atmospheric pressure chemical ionization (APCI).

Disulfide **4**, 2-Nitro-5-(octyldisulfaneyl)benzoic acid: A flame-dried round-bottom flask was charged with 5,5’-Dithiobis-(2-nitrobenzoic acid) (DTNB) (1.19 g, 3.00 mmol, 1.00 eq.), octanethiol (0.52 mL, 3.0 mmol, 1.0 eq.) and CH_2_Cl_2_ (25 mL) under an argon atmosphere. Et_3_N (1.5 mL, 11 mmol, 3.5 eq.) was added and the reaction was stirred for 2 h. The solvent was removed in vacuo before quenching with 1 M HCl (20 mL) and extraction with EtOAc (3 × 20 mL). The organics were dried over MgSO_4_, filtered and concentrated in vacuo. The crude product was purified by column chromatography (SiO_2_, CH_2_Cl_2_:MeOH:AcOH 97:2:1) and freeze dried to yield disulfide **4** as an orange solid (440 mg, 43% yield). ^**1**^**H NMR** (400 MHz, (CD_3_)_2_SO): δ 8.03 (d, *J* = 8.5 Hz, 1 H), 7.91 (d, *J* = 2.0 Hz, 1 H), 7.87 (dd, *J* = 8.5 Hz, 2.0 Hz, 1 H), 2.83 (t, *J* = 7.0 Hz, 2 H), 1.62–1.54 (m, 2 H), 1.34–1.19 (m, 10 H), 0.82 (t, *J* = 7.0 Hz, 3 H); ^**13**^**C NMR** (101 MHz, (CD_3_)_2_SO): δ 165.7, 145.7, 144.6, 128.9, 128.2, 125.7, 124.9, 38.2, 31.2, 28.5, 28.3, 27.6, 22.0, 13.9; **IR** ṽ_max_ (film)/cm^−1^: 2925, 2854, 1710, 1610, 1568, 1526; **HRMS** (ESI + , m/z): [M + Na]^+^ calcd. for C_15_H_21_O_4_NNaS_2_, 366.0810; found, 366.0803.

Thiol **3**, 5-mercapto-2-nitrobenzoic acid: A two-necked round-bottom flask equipped with a dropping funnel and an exit needle was charged with a suspension of 5,5′-dithiobis-(2-nitrobenzoic acid) (420 mg, 1.06 mmol, 1.00 eq) in EtOH (80% v/v in H_2_O, 5.0 mL). NaBH_4_ (320 mg, 8.50 mmol, 8.00 eq.) in distilled H_2_O (2.0 mL) was added dropwise to the suspension at 0 °C. The reaction was stirred at room temperature until gas evolution subsided and was then diluted with EtOH (5 mL) and H_2_O (5 mL) before acidification with 1 M HCl (10 mL). The mixture was extracted with CH_2_Cl_2_ (3 × 20 mL) and the combined organic layers dried over MgSO_4_, filtered, and concentrated in vacuo to yield thiol **3** as an orange solid (405 mg, 96% yield). NMR spectra were recorded in (CD_3_)_2_SO and in a 500 mM Tris buffer solution in D_2_O to completely avoid oxidation. This solution was prepared by dissolution of Trizma^®^ base (0.303 g, 2.50 mmol) in D_2_O (5.0 mL) before adjustment to pH* ~9 with DCl (0.5 M in D_2_O). Dissolution of **3** (20 mg, 0.10 mmol) in this buffered solution resulted in a solution of pH* ~8. ^**1**^**H NMR** (400 MHz, (CD_3_)_2_SO): δ 8.03 (d, *J* = 8.5 Hz, 1 H), 7.93 (br. s, 1 H), 7.86 (br. d, *J* = 8.5 Hz, 1 H); ^**1**^**H NMR** (400 MHz, 500 mM Tris in D_2_O, pH* ~8): δ 7.81 (d, *J* = 8.5 Hz, 1 H), 7.38 (dd, *J* = 9.0 Hz, 2.0 Hz, 1 H), 7.26 (d, *J* = 2.0 Hz, 1 H); ^**13**^**C NMR** (101 MHz, (CD_3_)_2_SO): δ 165.5, 146.4, 141.9, 129.1, 129.0, 126.7, 125.3; ^**13**^**C NMR** (101 MHz, 500 mM Tris in D_2_O, pH* ~8): δ 176.0, 163.4, 137.2, 135.9, 132.5, 130.2, 123.8; **IR** ṽ_max_ (film)/cm^−1^: 1704, 1642, 1604, 1570, 1510; **HRMS** (ESI-, m/z): [M-H]^−^ calcd. for C_7_H_4_O_4_NS, 197.98665; found, 197.98660.

Disulfide **5**, 1,2-dioctyldisulfane: A round-bottom flask was charged with a solution of octanethiol (0.47 mL, 2.7 mmol, 1.0 eq.) in Et_2_O (9.0 mL). To the solution was added an aqueous solution of H_2_O_2_ (50 wt%, 160 μL, 1.0 eq.) and NaI (tip of spatula). After stirring for 10 min the reaction was quenched with sat. aq. Na_2_S_2_O_3_ (5 mL) and extracted with Et_2_O (3 × 5 mL). The combined organics were dried over MgSO_4_, filtered, and concentrated in vacuo to yield disulfide **5** as a clear oil (314 mg, 79% yield). ^**1**^**H NMR** (400 MHz, CDCl_3_): δ 2.68 (t, *J* = 7.5 Hz, 4 H), 1.70–1.63 (m, 4 H), 1.41–1.27 (m, 20 H), 0.88 (t, *J* = 7.0 Hz, 6 H); ^**13**^**C NMR** (101 MHz, CDCl_3_): δ 39.4, 32.0, 29.4, 29.4, 29.3, 28.7, 22.8, 14.2; **IR** ṽ_max_ (film)/cm^−1^: 2955, 2923, 2853; **HRMS** (APCI, m/z): [M + H]^+^ calcd. for C_16_H_35_S_2_, 291.21747; found, 291.21737.

Preparation of 500 mM Tris buffer for biphasic reactions and for physical characterisation of **4**: Per 50 mL Tris buffer: Trizma^®^ base obtained from Sigma-Aldrich (3.029 g, 25.00 mmol) was dissolved in MilliQ H_2_O (approx. 45 mL). The solution was adjusted to pH ~7.8–8.0 with 12 M HCl (unless otherwise indicated in procedure) and the volume made up to 50 mL with MilliQ H_2_O. The buffer solution was stored at 4 °C before use. In general, a 25 mM solution of DTNB **2** prepared in this buffer would be pH ~7.5. Any subsequent adjustment of pH is indicated in procedures.

### Dynamic light scattering

The size of aggregates of **4** in 500 mM Tris buffer at pH 7.5 with variation in concentration of **4** was measured by dynamic light scattering. A 50 mM solution of disulfide **4** was prepared by dissolution of disulfide **4** (86 mg, 0.25 mmol) in Tris buffer (500 mM, pH 7.8–8.0, 5.0 mL). The solution was adjusted to pH 7.5 with a few drops of 1 M HCl. Subsequent twofold serial dilutions were made with a Tris buffer solution (500 mM, pH 7.5) to a concentration of 0.39 mM. Analyses were performed using a Malvern Zetasizer Nano ZEN5600 model system recording particle and molecule size. Instrument control and data processing were performed using Zetasizer software. Disposable plastic cuvettes were used with 1.0 mL of sample solution, which was filtered through a 200 nm filter immediately before measurement. Measurements were performed using an equilibrated heating probe at 25 °C, setting the following parameters for 500 mM Tris buffer (calculated internally using the software’s complex solvent builder): refractive index = 1.339, viscosity = 1.0780 mPa s. Three repetitions of ten measurements were taken for every concentration. Data are shown in Supplementary Figures 1–3.

### Critical micelle concentration

The critical micelle concentration of **4** in 500 mM Tris buffer at pH 7.5 was calculated by measuring the surface tension of solutions of different concentration of **4**; the critical micelle concentration is the calculated point at which surface tension no longer decreases with increasing concentration of **4**. Prior to buffer preparation, Trizma^®^ base was recrystallised twice from 20% EtOH/H_2_O, and dried over P_2_O_5_ overnight. A 40 mM solution of disulfide **4** was prepared by dissolution of disulfide **4** (275 mg, 0.8 mmol) in Tris buffer (500 mM, pH 7.9, 20.0 mL). The solution was adjusted to pH ~7.5 with a very small quantity of 12 M HCl. Subsequent twofold serial dilutions were made with a Tris buffer solution (500 mM, pH 7.5) to a concentration of 4.9 × 10^−3^ mM. The surface tension of each solution was measured using a Kruss K10ST tensiometer, calibrated with the surface tension of pure water. Measurements were performed at 25 °C and each solution was measured three times. All measurements were taken on the same day. Data are shown in Supplementary Figure 4.

### Kinetic analyses of biphasic reactions

The concentrations of all UV active reaction species in aliquots removed from reaction were monitored by UPLC using an external standard. The external standard solution was first prepared by the dissolution of 3-methyl-2-nitrobenzoic acid (100 mg, 0.55 mmol) in DMSO (10 mL). 2.5 mL of this solution was diluted with MilliQ H_2_O to a total volume of 500 mL in a volumetric flask, giving an overall concentration of 0.05 mg mL^−1^ (0.276 mM). To this standard solution was added maleimide (120 mg per 20 mL), which acted as a quench for the reaction. To monitor the reactions (unless otherwise specified) a small volume of the reaction mixture (aqueous phase) was withdrawn at specified time points by plastic syringe. A 25 μL aliquot of this withdrawn solution was then measured by microsyringe and placed into an MS vial, before quenching with 1.0 mL of external standard solution containing maleimide. One microliter of the resultant solution was the standard injection volume for analysis by UPLC. Chromatograms of each resultant sample were obtained using a Waters Acquity ultra performance liquid chromatography (UPLC) H-Class system with photodiode array (PDA) detector. Instrument control and data processing were performed using Empower software. An Acquity UPLC BEH C18 column (130 Å, 1.7 µm, 2.1 mm × 50 mm) was used. A mixture of H_2_O:MeCN:5% TFA in H_2_O with a gradient of 93:2:5 → 0:95:5 over 5 min was used as mobile phase. Peak areas were integrated at a wavelength of 215 nm. Calibration of reaction components showing linear fitting was obtained (Supplementary Figures 5–7) to determine component concentrations.

### Control and seeded reactions

Control reaction preparation: 5,5′-Dithiobis-(2-nitrobenzoic acid) (DTNB) (**2**) (119 mg, 300 µmol) was dissolved by sonication in 12 mL Tris buffer (500 mM, pH 7.8–8.0) to reach pH ~7.5. Any necessary adjustment was made with a few drops of 12 M HCl.

Seeded (15 mM) reaction preparation: 5,5′-Dithiobis-(2-nitrobenzoic acid) (DTNB) (**2**) (60 mg, 0.15 mmol) was dissolved by sonication in 6 mL Tris buffer (500 mM, pH 7.8–8.0). To this solution was added disulfide **4** (31 mg, 90 µmol) with sonication for dissolution, to reach a pH of ~7.5 (matching the pH of the control). Any necessary adjustment was made with a few drops of 12 M HCl.

Five milliliters of each solution was placed in a 10 mL round bottomed flask and stirring was started at 100 rpm. Octanethiol **1** (22 µL, 0.13 mmol, 1.0 eq. OR 174 μL, 1.00 mmol, 8.00 eq.) was placed dropwise onto the surface of the solution, and the beginning of this addition was considered time zero. The data from these experiments is shown in Fig. 3b–e. The extraction of initial rates for these reactions is shown in Supplementary Figures 8 and 9.

### Additional seeding experiments

Reactions at lower concentration of seed were performed analogously to the above control and seeded reactions. For these experiments, however, no additional pH adjustment was made after dissolution of the disulfides in Tris buffer (500 mM, pH 7.8–8.0) to avoid significant difference in reaction volume between experiments. Thus, pH is likely to have varied slightly between experiments conducted with different quantity of the seeded product **4**. Seeding reactions conducted with 0.1 mM and 1 mM disulfide **4** were conducted by first preparing a stock solution (2.5 mM) of disulfide **4** in Tris buffer (500 mM, pH 7.8–8.0). The data from these experiments is shown in Fig. 3f, g. The extraction of initial rates for these reactions is shown in Supplementary Figure 10.

### Fuelled experiments

5,5′-Dithiobis-(2-nitrobenzoic acid) (DTNB) (**2**) (119 mg, 300 µmol) was dissolved by sonication in 12 mL Tris buffer (500 mM, pH 7.8–8.0) to reach pH ~7.5. Any necessary adjustment was made with a few drops of 12 M HCl. Five milliliters of this solution was placed in a 10 mL round bottomed flask and stirring was started at 100 rpm.

Preparation of oxidant solution: 36 μL of an aqueous solution of H_2_O_2_ (50% wt.) was added to 64 μL of MilliQ H_2_O. To this was added an additional 0.45 mL of MilliQ H_2_O. The solution was placed into a 1.0 mL plastic syringe and the solution pushed to the needle tip before placing in the syringe pump assembly and tightening the assembly. Excess solution from the needle tip was wiped away before lowering into the aqueous solution. The syringe pump was then turned on and run at the initial rate indicated. Octanethiol **1** was added dropwise by microsyringe onto the top of the solution momentarily after starting the pump. The beginning of this addition was considered time zero. The fuel addition was stopped or the rate altered at the time points indicated in Fig. 5 by dashed grey lines.

## Supplementary information

Supplementary Information

## Data Availability

Data are available upon request from the authors.

## References

[1] Schrodinger, E. *What is life? The Physical Aspect of the Living Cell* (Cambridge University Press, Cambridge, 1944).

[2] Pross, A. *What is Life? How chemistry becomes biology* (Oxford University Press, Oxford, 2016).

[3] Schwille P (2017). How simple could life be?. Angew. Chem. Int. Ed..

[4] Ashkenasy G, Hermans TM, Otto S, Taylor AF (2017). Systems chemistry. Chem. Soc. Rev..

[5] van Rossum SAP, Tena-Solsona M, van Esch JH, Eelkema R, Boekhoven J (2017). Dissipative out-of-equilibrium assembly of man-made supramolecular materials. Chem. Soc. Rev..

[6] Grzybowski BA, Huck WTS (2016). The nanotechnology of life-inspired systems. Nat. Nanotechnol..

[7] Ragazzon G, Prins LJ (2018). Energy consumption in chemical fuel-driven self-assembly. Nat. Nanotechnol..

[8] Boekhoven J, Hendriksen WE, Koper GJM, Eelkema R, van Esch JH (2015). Transient self-assembly of active materials fueled by a chemical reaction. Science.

[9] Erbas-Cakmak S (2017). Rotary and linear molecular motors driven by pulses of a chemical fuel. Science.

[10] Fletcher SP, Dumur F, Pollard MM, Feringa BL (2005). A reversible, unidirectional molecular rotary motor driven by chemical energy. Science.

[11] Collins BSL, Kistemaker JCM, Otten E, Feringa BL (2016). A chemically powered unidirectional rotary molecular motor based on a palladium redox cycle. Nat. Chem..

[12] Koumura N (1999). Light-driven monodirectional molecular rotor. Nature.

[13] Maiti S, Fortunati I, Ferrante C, Scrimin P, Prins LJ (2016). Dissipative self-assembly of vesicular nanoreactors. Nat. Chem..

[14] Tena-Solsona M, Wanzke C, Riess B, Bausch AR, Boekhoven J (2018). Self-selection of dissipative assemblies driven by primitive chemical reaction networks. Nat. Commun..

[15] He X (2012). Synthetic homeostatic materials with chemo-mechano-chemical self-regulation. Nature.

[16] Semenov SN (2016). Autocatalytic, bistable, oscillatory networks of biologically relevant organic reactions. Nature.

[17] Semenov SN (2015). Rational design of functional and tunable oscillating enzymatic networks. Nat. Chem..

[18] Bissette AJ, Fletcher SP (2013). Mechanisms of autocatalysis. Angew. Chem. Int. Ed..

[19] Kosikova T, Philp D (2017). Exploring the emergence of complexity using synthetic replicators. Chem. Soc. Rev..

[20] Duim H, Otto S (2017). Towards open-ended evolution in self-replicating molecular systems. Beilstein J. Org. Chem..

[21] Higgs PG (2017). Chemical evolution and the evolutionary definition of life. J. Mol. Evol..

[22] Sadownik JW, Mattia E, Nowak P, Otto S (2016). Diversification of self-replicating molecules. Nat. Chem..

[23] Saghatellan A, Yokobayashi Y, Soltani K, Ghadiri MR (2001). A chiroselective peptide replicator. Nature.

[24] von Kiedrowski G (1986). A self‐replicating hexadeoxynucleotide. Angew. Chem. Int. Ed..

[25] Lee DH, Granja JR, Martinez JA, Severin K, Ghadiri MR (1996). A self-replicating peptide. Nature.

[26] Tjivikua T, Ballester P, Rebek J (1990). A self-replicating system. J. Am. Chem. Soc..

[27] Bachmann PA, Luisi PL, Lang J (1992). Autocatalytic self-replicating micelles as models for prebiotic structures. Nature.

[28] Bissette AJ, Odell B, Fletcher SP (2014). Physical autocatalysis driven by a bond-forming thiol–ene reaction. Nat. Commun..

[29] Carnall JMA (2010). Mechanosensitive self-replication driven by self-organization. Science.

[30] Colomer I, Morrow SM, Fletcher SP (2018). A transient self-assembling self-replicator. Nat. Commun..

[31] Zepik HH, Blöchliger E, Luisi PL (2001). A chemical model of homeostasis. Angew. Chem. Int. Ed..

[32] Luisi PL (2014). The minimal autopoietic unit. Orig. Life. Evol. Biosph..

[33] Buhse T, Pimienta V, Lavabre D, Micheau JC (1997). Experimental evidence of kinetic bistability in a biphasic surfactant system. J. Phys. Chem. A.

[34] Stano P, Luisi PL (2010). Achievements and open questions in the self-reproduction of vesicles and synthetic minimal cells. Chem. Commun..

[35] Szostak JW, Bartel DP, Luisi PL (2001). Synthesizing life. Nature.

[36] Kurihara K (2011). Self-reproduction of supramolecular giant vesicles combined with the amplification of encapsulated DNA. Nat. Chem..

[37] Ortega-arroyo J, Bissette AJ, Kukura P, Fletcher SP (2016). Visualization of the spontaneous emergence of a complex, dynamic, and autocatalytic system. Proc. Natl Acad. Sci. USA.

[38] Post E, Bissette A, Fletcher SP (2018). Self-reproducing micelles coupled to a secondary catalyst. Chem. Commun..

[39] Newsholme, E. A. *Regulation in Metabolism* (John Wiley & Sons, London, 1973).

[40] Curi R (2016). Regulatory principles in metabolism - then and now. Biochem. J..

[41] Nguyen R, Allouche L, Buhler E, Giuseppone N (2009). Dynamic combinatorial evolution within self-replicating supramolecular assemblies. Angew. Chem. Int. Ed..

[42] Singh, R. & Whitesides, G. M. Thiol-disulfide interchange. in *The Chemistry of Functional Groups, Supplement S: The Chemistry of Sulphur-Containing Functional Groups* (eds Patai, S. & Rappoport, Z.) 633–658 (John Wiley & Sons, Chichester, 1993).

