# Supplementary material for: A chemically fuelled self-replicator
Source: Nat Commun. 2019 Mar 1;10:1011. doi: 10.1038/s41467-019-08885-9 (PMC6397266; doi:10.1038/s41467-019-08885-9)
Supplement: Supplementary file 1 — Supplementary Information [file 41467_2019_8885_MOESM1_ESM.pdf]

A Chemically Fuelled Self-Replicator

Morrow *et al.*

Supplementary Figures

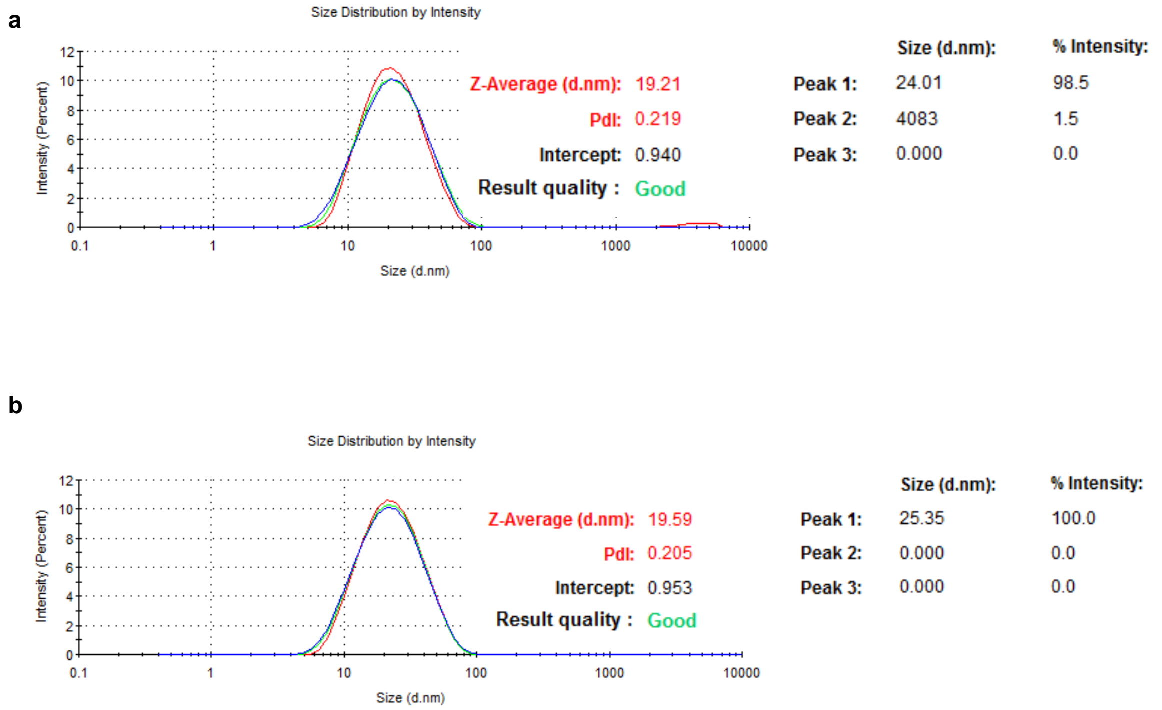

Supplementary Figure 1 Dynamic Light Scattering measurements Size distribution by intensity of **4** in 500 mM TRIS buffer pH 7.5. **a** 50 mM **b** 25 mM. Over the concentration range 50 – 3.125 mM aggregates of tight polydispersity were observed, from 15 – 20 nm in hydrodynamic diameter.

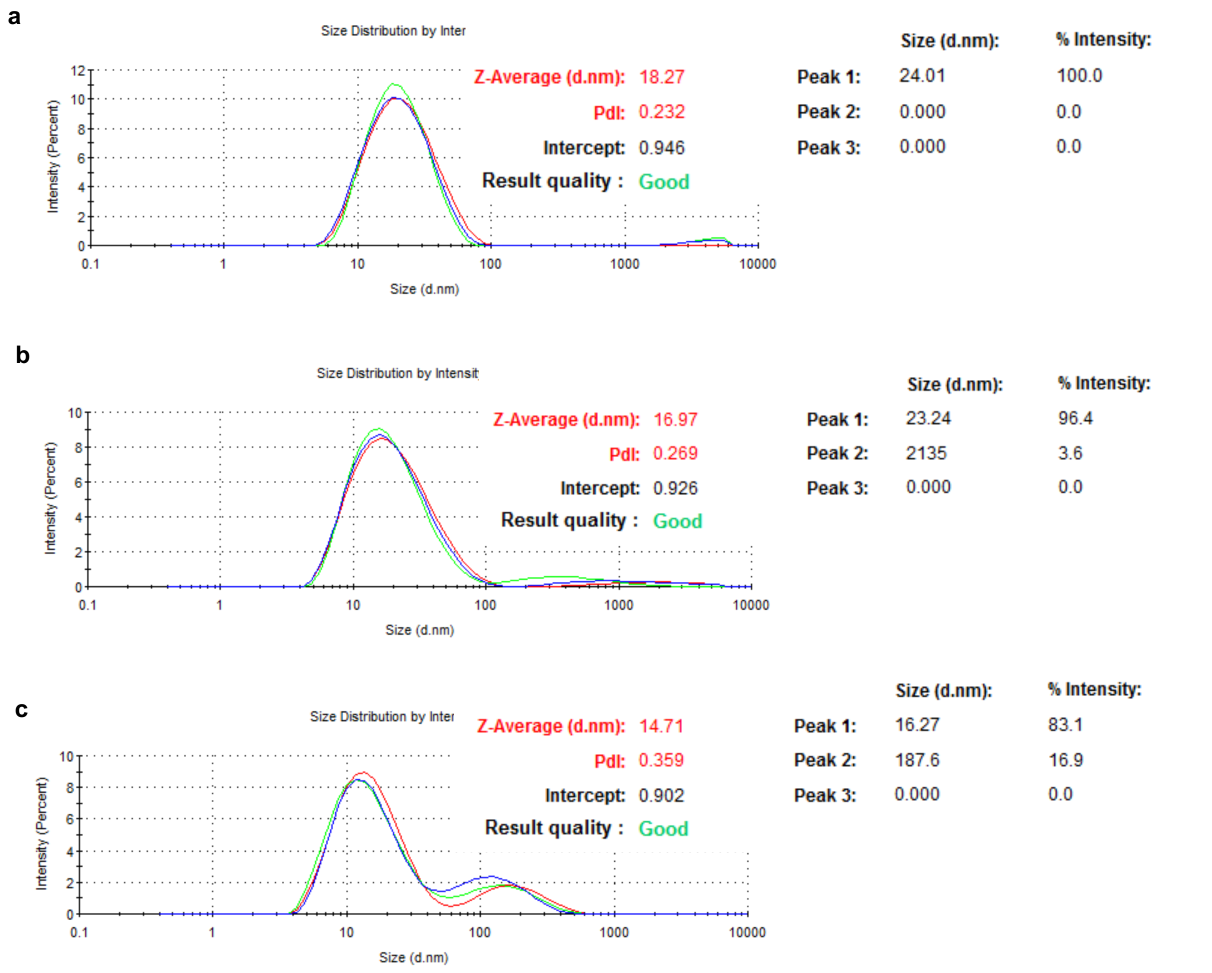

Supplementary Figure 2 Dynamic Light Scattering measurements Size distribution by intensity of **4** in 500 mM TRIS buffer pH 7.5 **a** 12.5 mM **b** 6.25 mM **c** 3.125 mM. Over the concentration range 50 – 3.125 mM aggregates of tight polydispersity were observed, from 15 – 20 nm in hydrodynamic diameter.

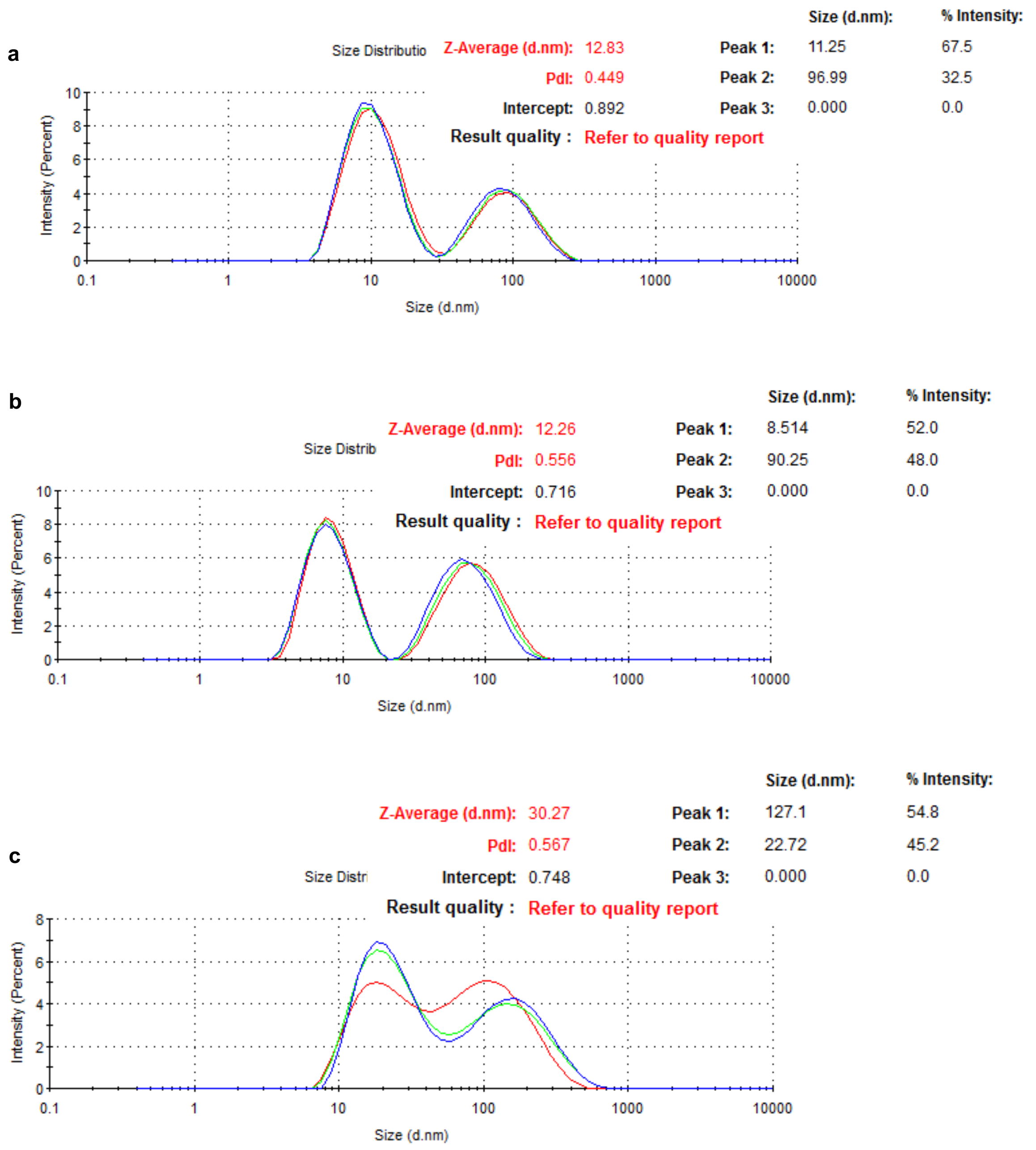

Supplementary Figure 3 Dynamic Light Scattering measurements Size distribution by intensity of **4** in 500 mM TRIS buffer pH 7.5 **a** 1.56 mM **b** 0.78 mM **c** 0.39 mM. In **a** and **b** a higher degree of polydispersity was observed compared to higher concentrations (Supplementary Figures 1,2). In **c**, count rate was significantly decreased, indicating reduced aggregation. When the sample concentration is too low, the scattering from the particles is weak and there is greater influence of noise from unwanted sources e.g. dust particles which may overwhelm the sample. This may explain the apparent increase in polydispersity. A lower count rate indicates reduced aggregation at low concentration.

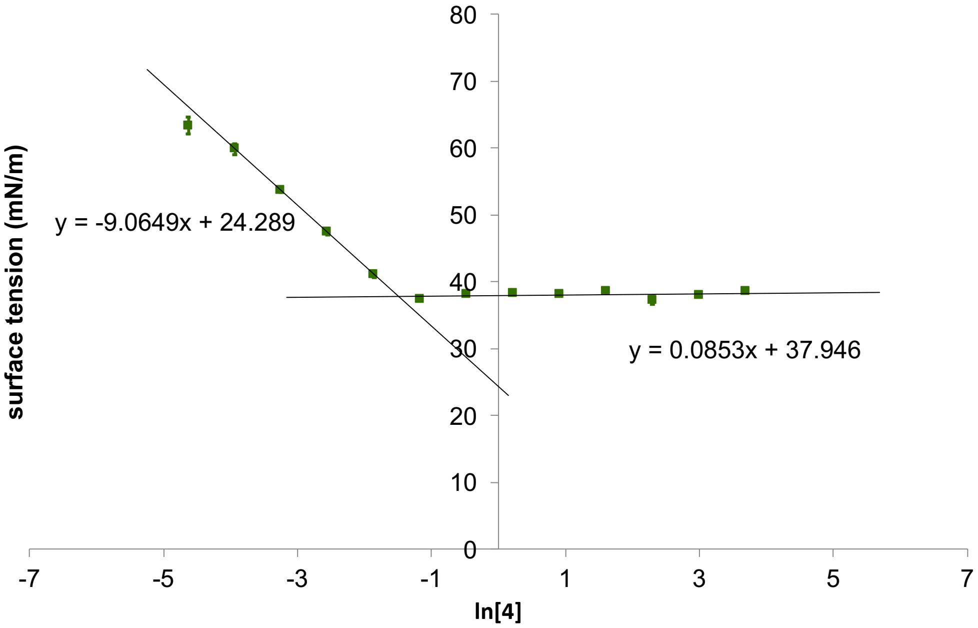

Supplementary Figure 4 Determination of critical micelle concentration by ring tensiometry A plot of surface tension, measured by ring tensiometry, against ln[**4**] with trendlines plotted both above and below the cmc. Each point is the average of three measurements. Error bars for the standard deviation of each measurement are plotted but are too small to be visible. At the cmc there is no longer a decrease in surface tension with concentration of surfactant. The intercept of the two lines therefore allows us to calculate the cmc. At the intercept:

$$x = -1.4925$$

$$[\mathbf{4}] = 0.22 \text{ mM}$$

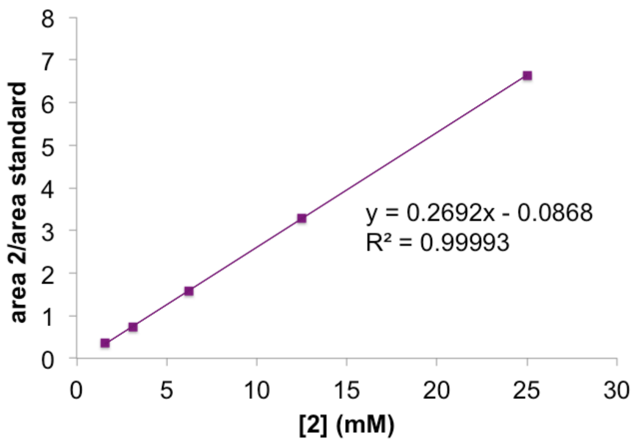

Supplementary Figure 5 UPLC calibration of 2. Solutions were prepared in 500 mM Tris buffer (prepared in Methods). Each point is the average of three measurements of the same sample. Error bars for the standard deviation of each point are plotted but are too small for observation.

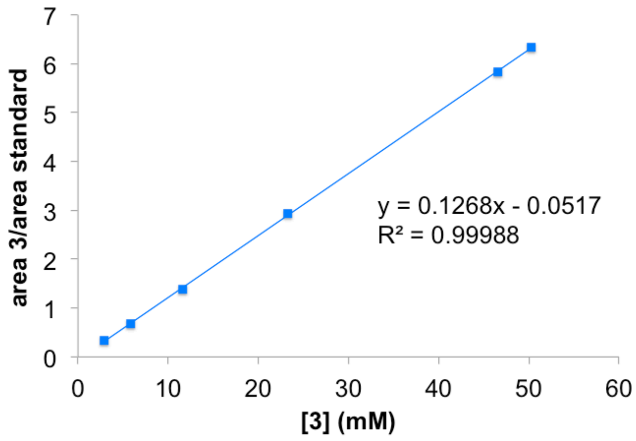

Supplementary Figure 6 UPLC calibration of 3. Solutions were prepared in 500 mM Tris buffer (prepared in Methods). Each point is the average of three measurements of the same sample. Error bars for the standard deviation of each point are plotted but are too small for observation.

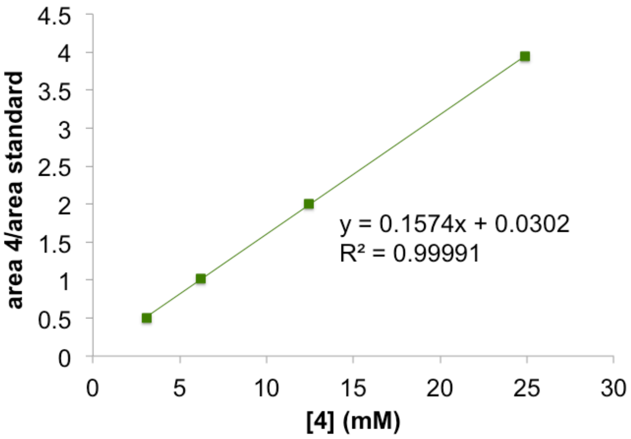

Supplementary Figure 7 UPLC calibration of 4. Solutions were prepared in 500 mM Tris buffer (prepared in Methods). Each point is the average of three measurements of the same sample. Error bars for the standard deviation of each point are plotted but are too small for observation.

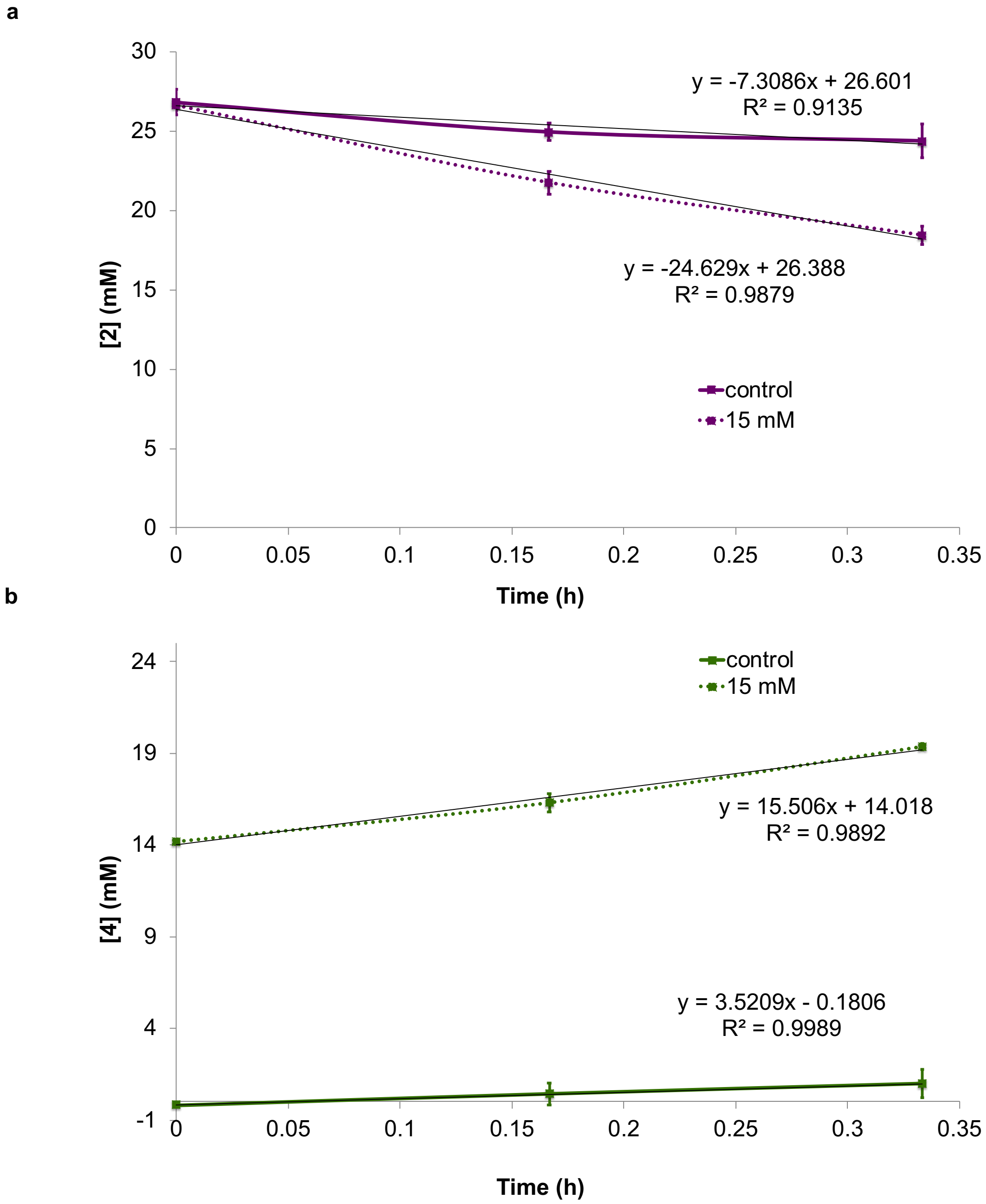

Supplementary Figure 8 Initial rate of control and seeded reactions Concentration of (**a**) **2** and (**b**) **4** over the course of the reaction conducted with 1.0 equivalent of octanethiol **1**, under control (solid line) and seeded (15 mM **4**, dotted line) conditions (early time points of Figures 3b,c). The concentration of reaction components was monitored by UPLC. Each plotted point is the average of three experiments and the error bars represent the standard deviation. Lines between points are drawn to guide the eye. Linear trendlines and their associated equations are displayed as a measure of initial rate.

a

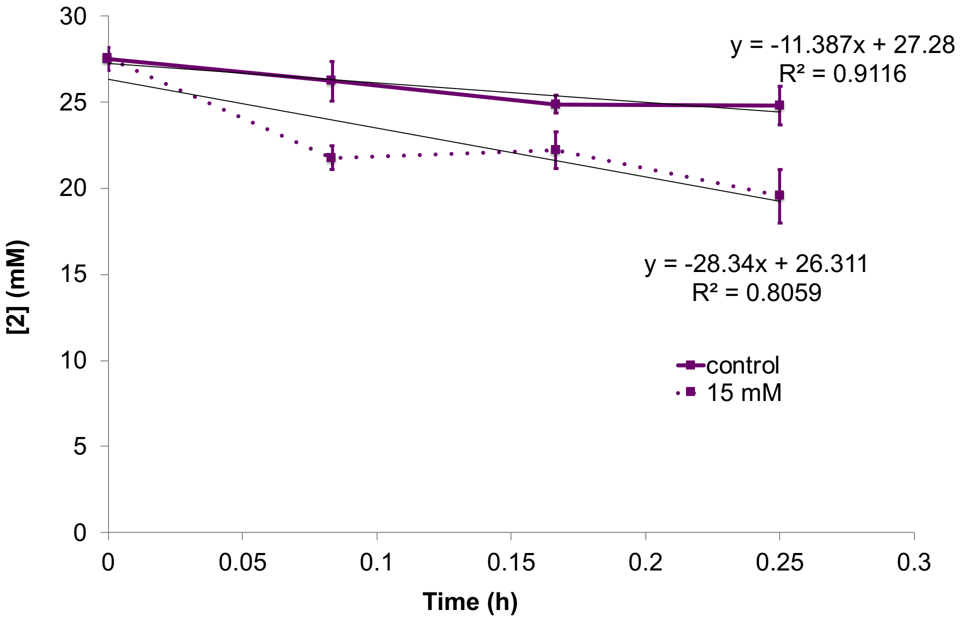

b

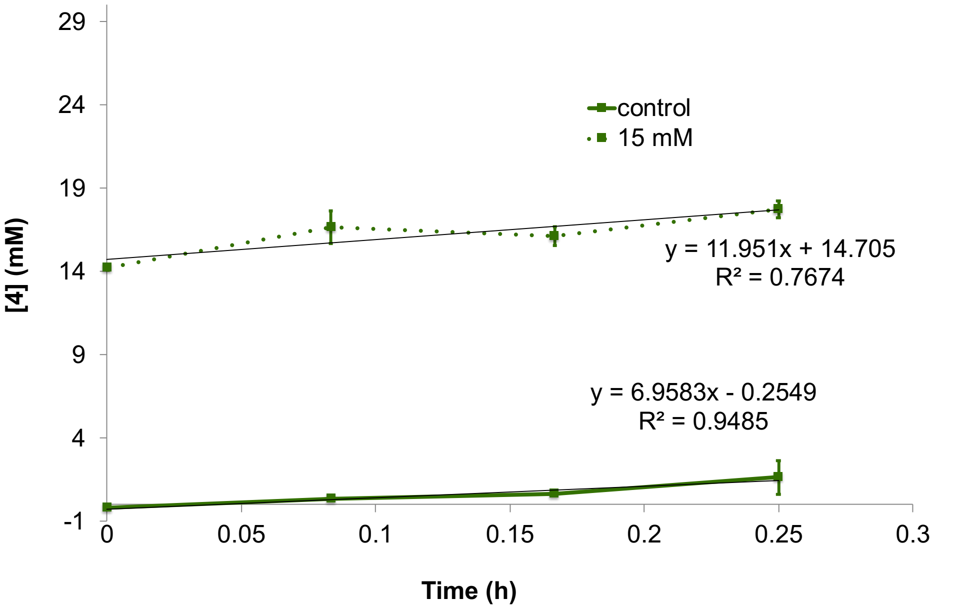

Supplementary Figure 9 Initial rate of control and seeded reactions Concentration of (**a**) **2** and (**b**) **4** over the course of the reaction conducted with 8.0 equivalents of octanethiol **1**, under control (solid line) and seeded (15 mM **4**, dotted line) conditions (early time points of Figures 3d,e). The concentration of reaction components was monitored by UPLC. Each plotted point is the average of three experiments and the error bars represent the standard deviation. Lines between points are drawn to guide the eye. Linear trendlines and their associated equations are displayed as a measure of initial rate.

a

b

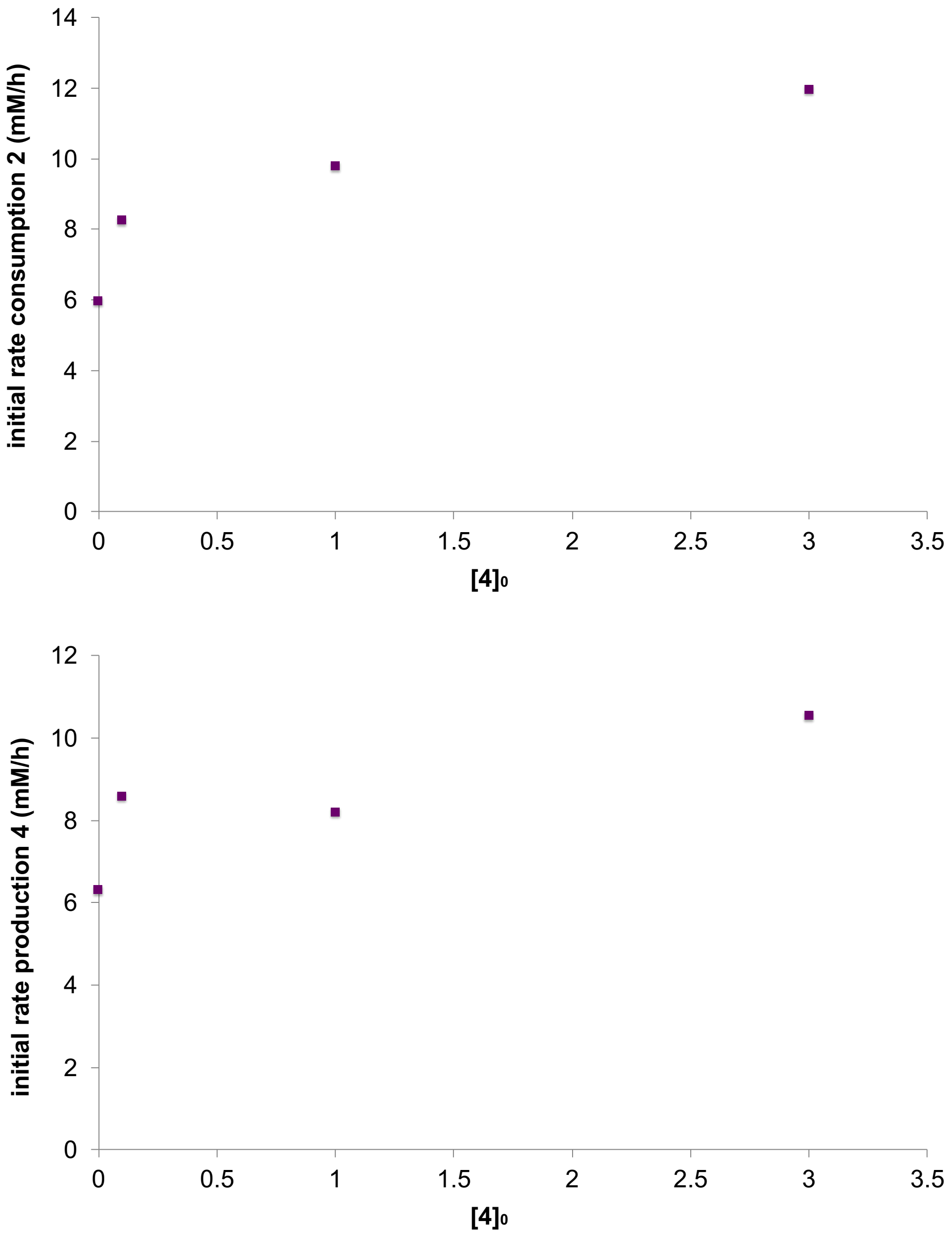

Supplementary Figure 10 Initial rate of control and seeded reactions a The measured initial rate of disulfide **2** consumption vs. the initial concentration of surfactant **4** (extracted from Figure 3f). **b** The measured initial rate of surfactant **4** production vs. the initial concentration of surfactant **4** (extracted from Figure 3g).

Supplementary Methods

Investigation into the destruction step

The second thiol-disulfide exchange, forming thermodynamic products **3** and **5**, occurs between hydrophobic thiol **1** and water-soluble surfactant **4**. Mechanistic investigation into this step was therefore important since it was possible that this step was also catalysed by the formation of aggregates.

To investigate the destruction step, the initial rates of the reactions in Supplementary Figure 11 with variation in [**4**]$_0$ both below and above the cmc were measured.

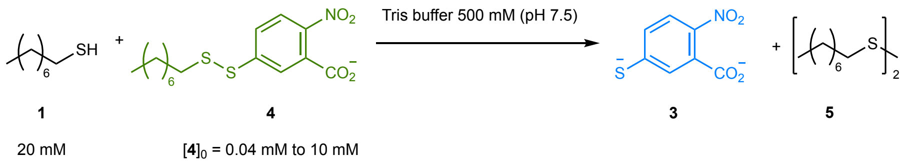

Supplementary Figure 11 Investigation into the destruction step Octanethiol **1** reacts with **4** to produce thiolate **3** and disulfide **5**. The initial rate of this reaction over a series of [**4**]$_0$ was measured to test if the reaction was catalysed by aggregates of **4**.

Solutions prepared for the measurement of critical micelle concentration (see Methods) with varying concentration of **4**, both below and above the measured cmc, were used for this study.

5 mL of each solution was placed in a 10 mL round bottomed flask and stirring was started at 100 rpm. Octanethiol **1** (20 µL, 0.10 mmol) was added dropwise by microsyringe onto the top of the solution and the start of this addition was considered time zero. The volume of aliquots, UPLC injection, and external standard were altered from the procedure described in Methods under Kinetic Analysis on account of the very low initial concentration of **4**. At each individual concentration, however, the procedure remained the same throughout the experiment.

The initial rates of the degradation of **4** and the production of **3** were extracted and plotted against [**4**]$_0$ (Supplementary Figures 12 and 13).

Within experimental error, the rate of production of **3** and the rate of degradation of **4** appeared to correlate linearly with the initial concentration of surfactant **4**; it did not appear that the formation of aggregates accelerated the reaction rate.

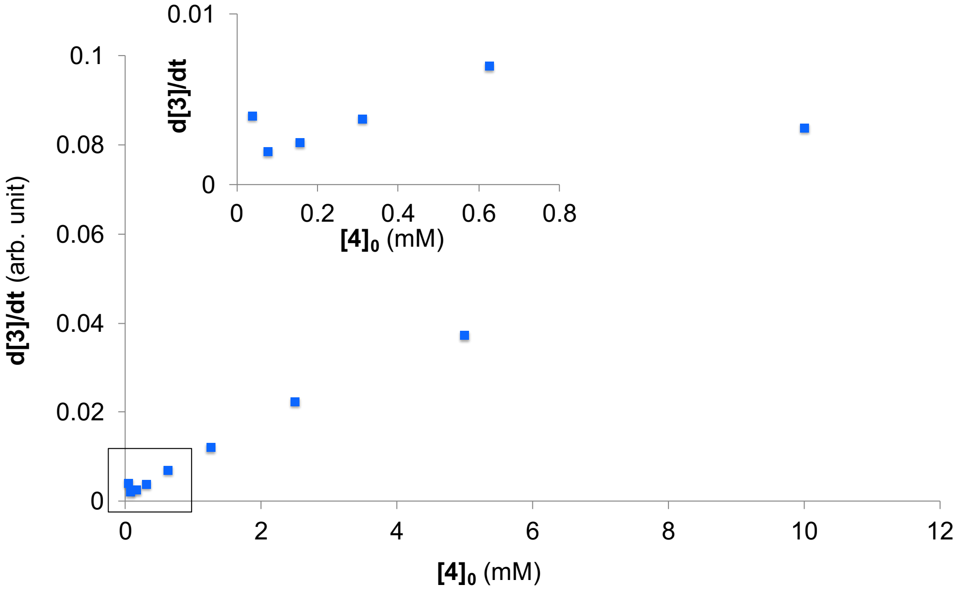

Supplementary Figure 12 Rate of surfactant destruction Initial rate of production of **3** with variation in [**4**]$_0$ for reaction in Supplementary Figure 11. The inset graph is the expanded data (from box in main graph) where [**4**]$_0$ approaches the measured cmc.

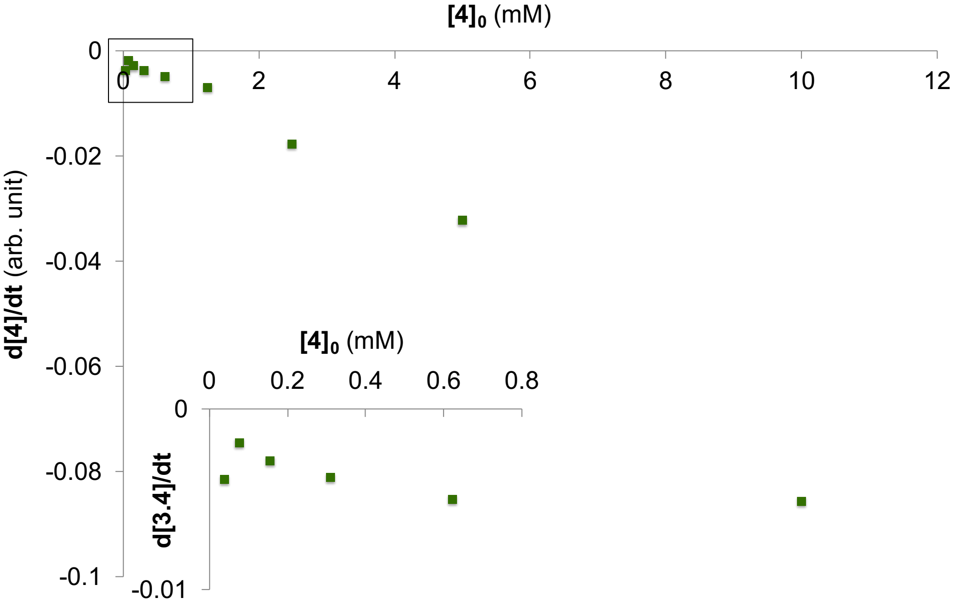

Supplementary Figure 13 Rate of surfactant destruction Initial rate of degradation of **4** with variation in $[\mathbf{4}]_0$ for reaction in Supplementary Figure 11. The inset graph is the expanded data (from box in main graph) where $[\mathbf{4}]_0$ approaches the measured cmc.

NMR spectra

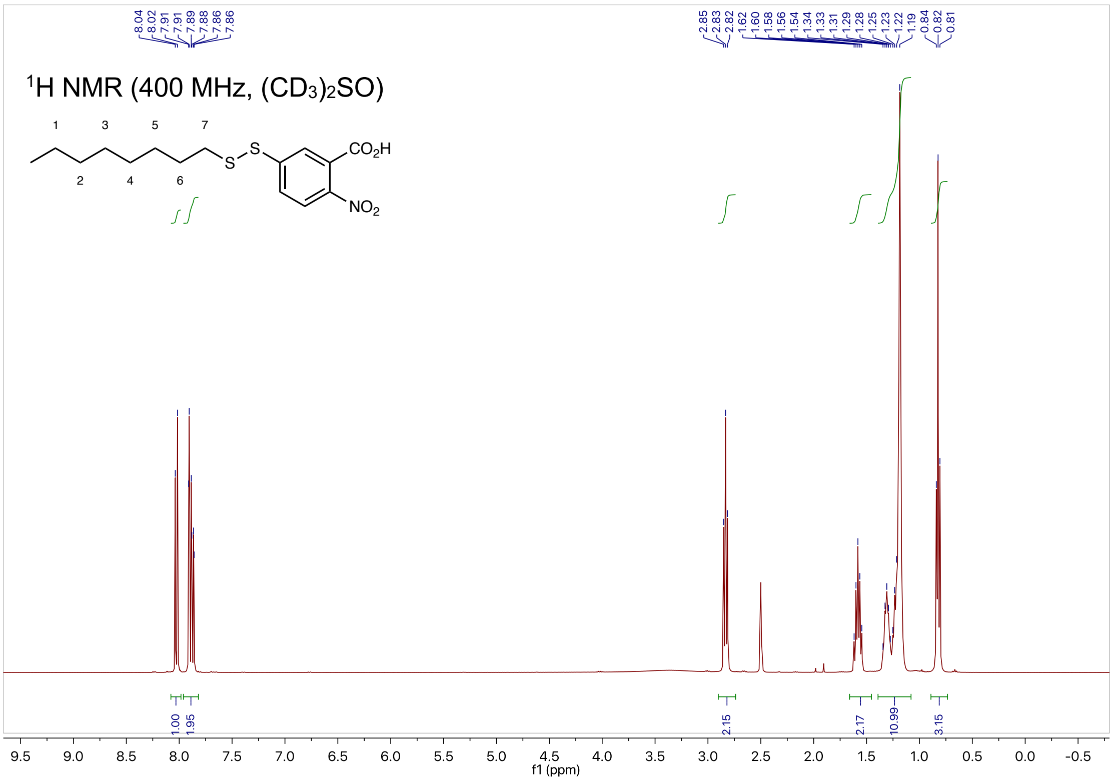

^{1}H NMR (400 MHz, $(CD_3)_2SO$): δ 8.03 (d, *J* = 8.5 Hz, 1H), 7.91 (d, *J* = 2.0 Hz, 1H), 7.87 (dd, *J* = 8.5 Hz, 2.0 Hz, 1H), 2.83 (t, *J* = 7.0 Hz, 2H), 1.62–1.54 (m, 2H), 1.34–1.19 (m, 10H), 0.82 (t, *J* = 7.0 Hz, 3H).

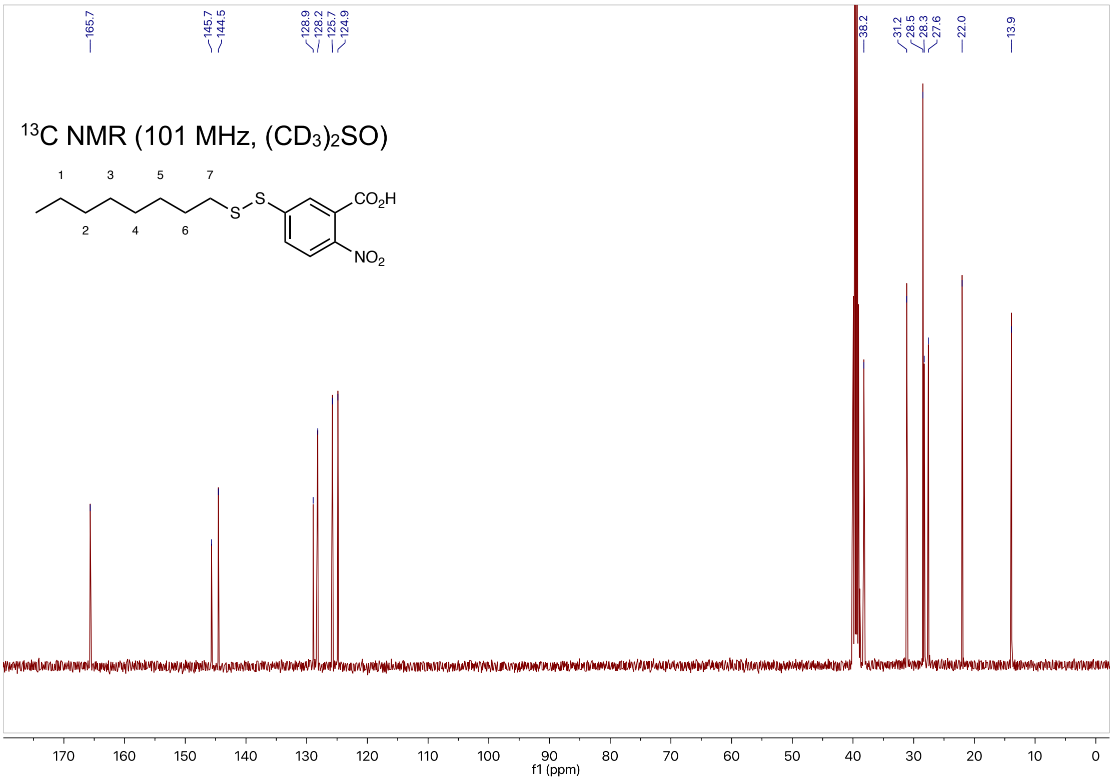

^{13}C NMR (101 MHz, $(CD_3)_2SO$): δ 165.7, 145.7, 144.6, 128.9, 128.2, 125.7, 124.9, 38.2, 31.2, 28.5, 28.3, 27.6, 22.0, 13.9.

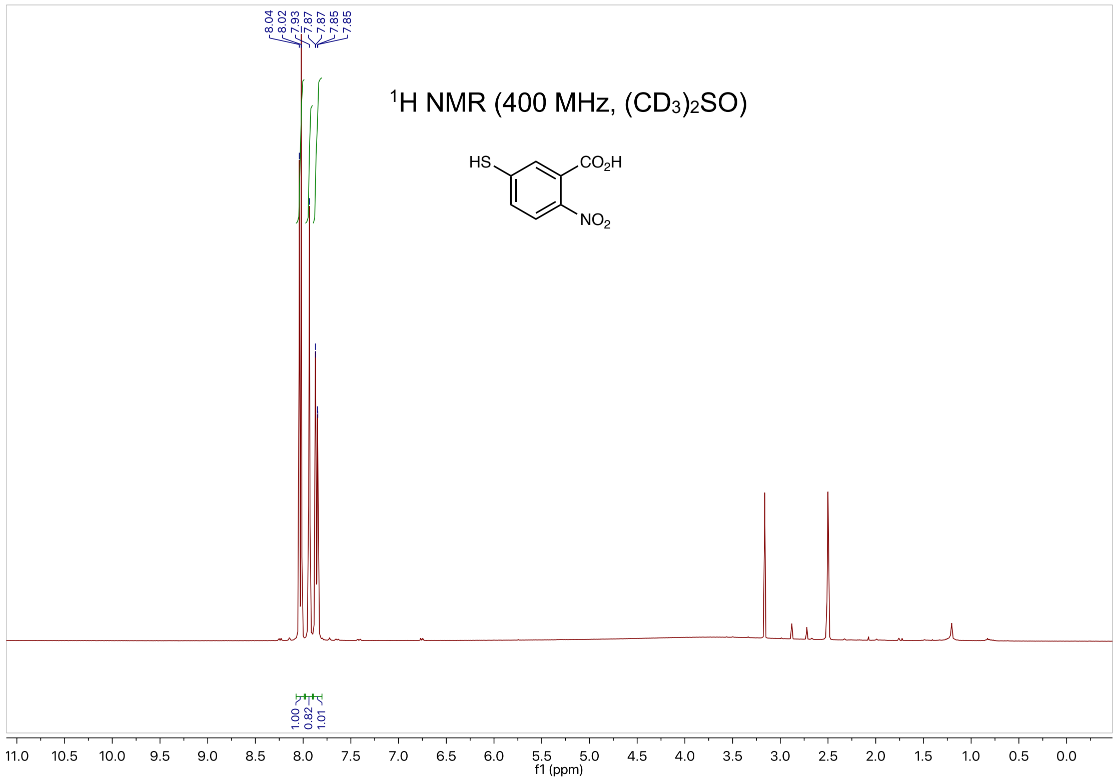

^{1}H NMR (400 MHz, $(CD_3)_2SO$): δ 8.03 (d, *J* = 8.5 Hz, 1H), 7.93 (br. s, 1H), 7.86 (br. d, *J* = 8.5 Hz, 1H).

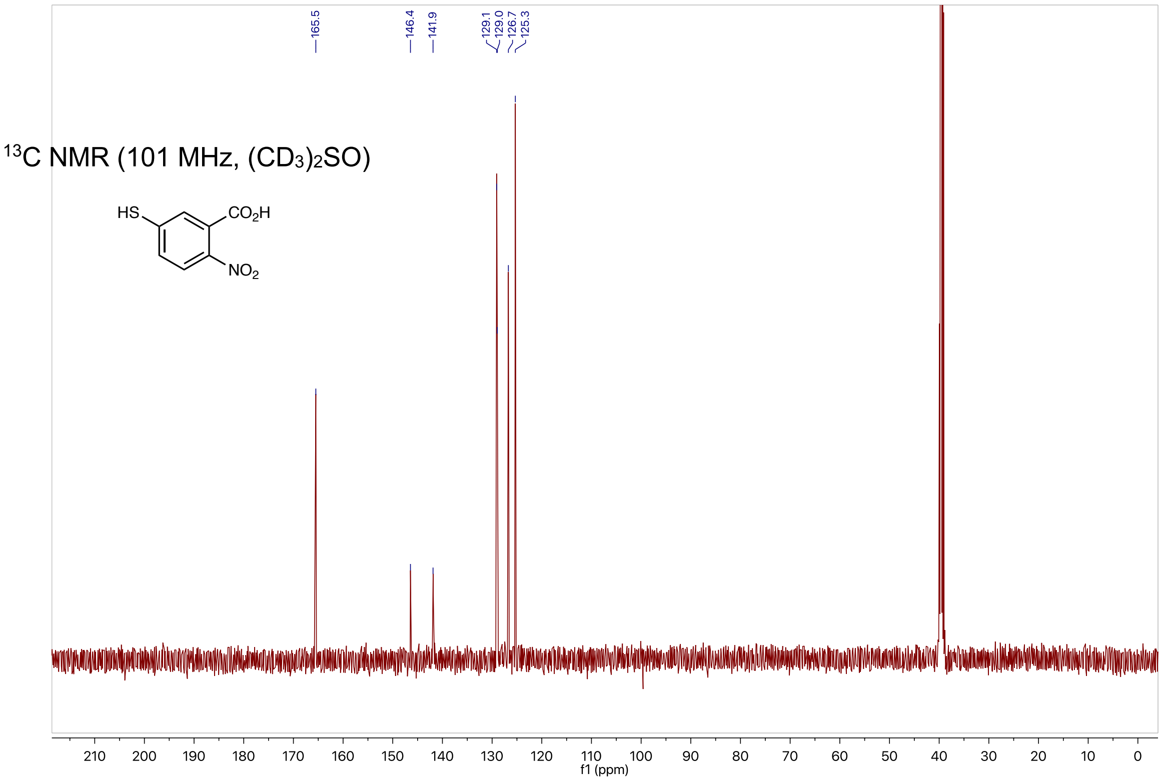

^{13}C NMR (101 MHz, $(CD_3)_2SO$): δ 165.5, 146.4, 141.9, 129.1, 129.0, 126.7, 125.3.

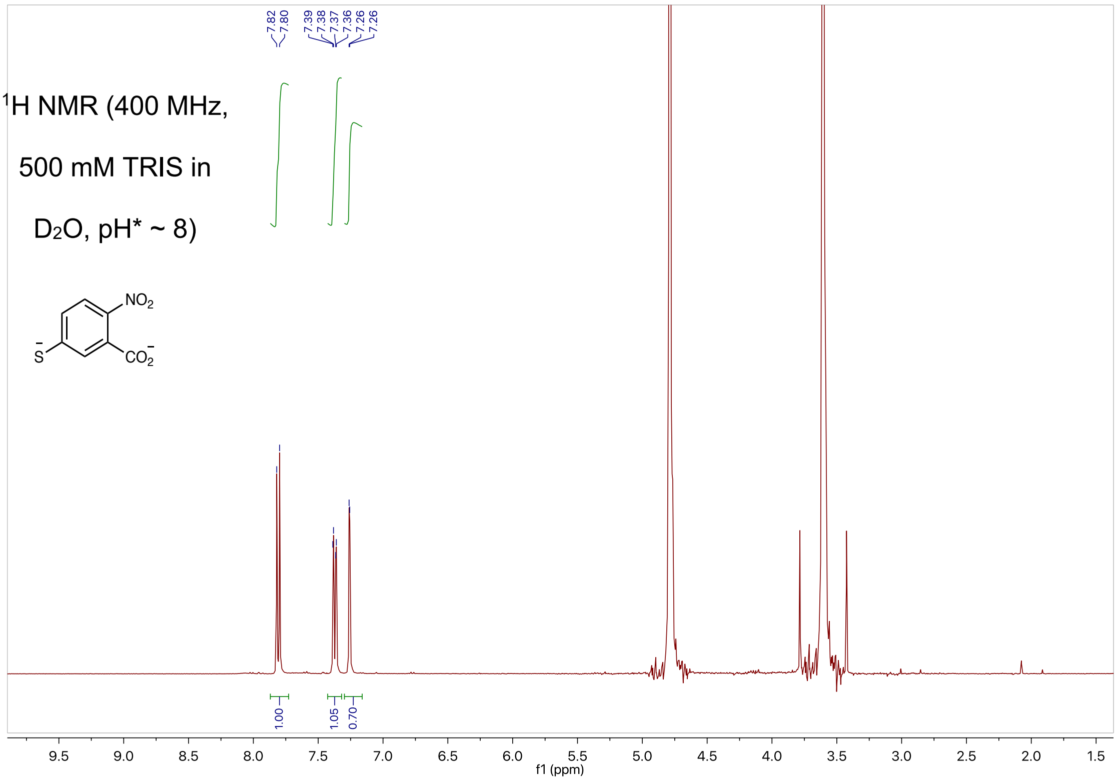

^{1}H NMR (400 MHz, 500 mM TRIS in D_2O, pH* ~ 8): δ 7.81 (d, *J* = 8.5 Hz, 1H), 7.38 (dd, *J* = 9.0 Hz, 2.0 Hz, 1H), 7.26 (d, *J* = 2.0 Hz, 1H).

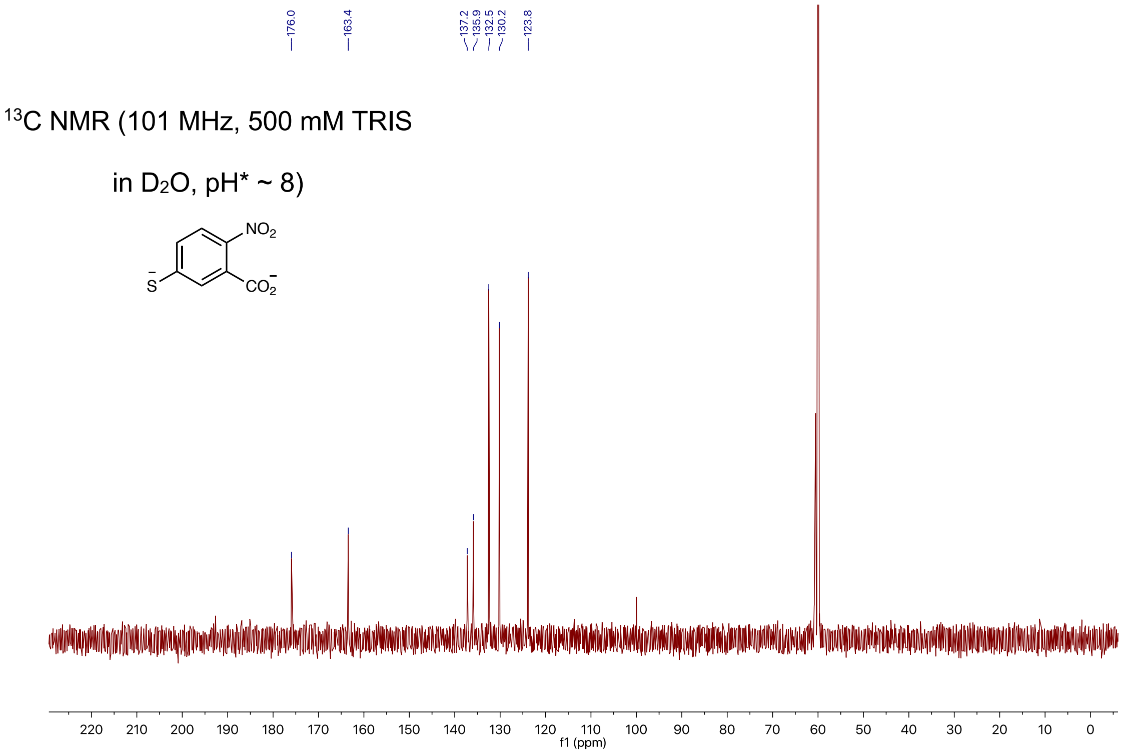

^{13}C NMR (101 MHz, 500 mM TRIS in D_2O, pH* ~ 8): δ 176.0, 163.4, 137.2, 135.9, 132.5, 130.2, 123.8.

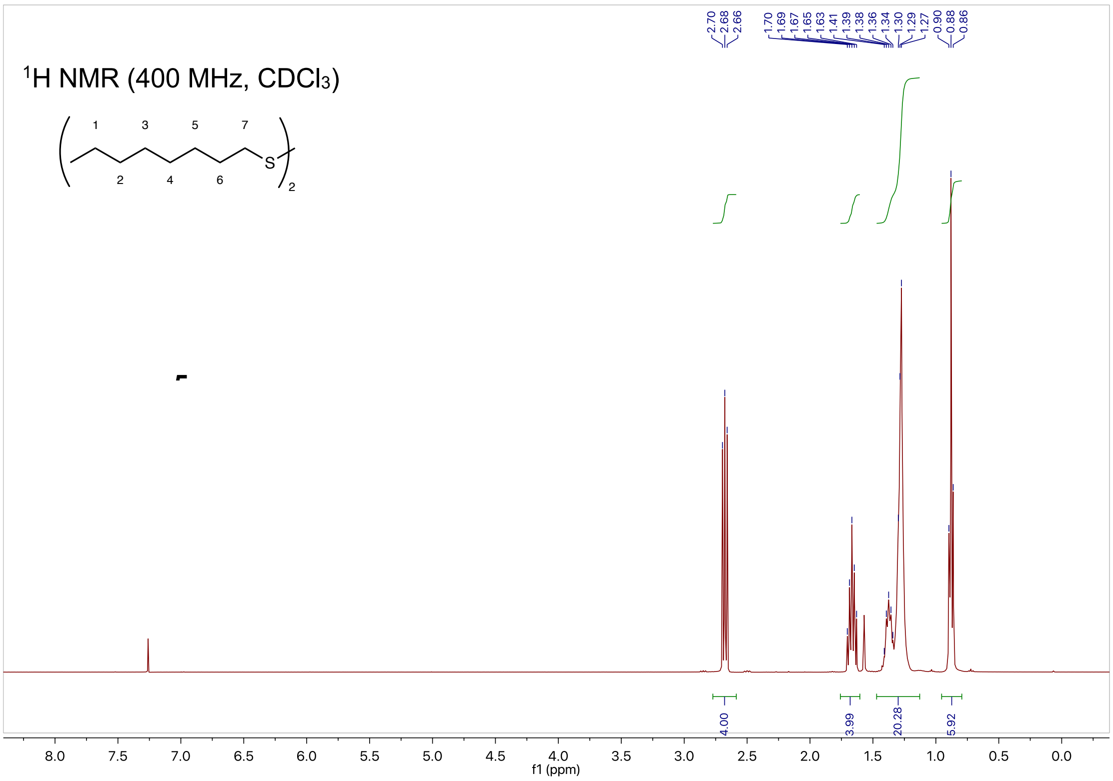

^{1}H NMR (400 MHz, $CDCl_3$): δ 2.68 (t, *J* = 7.5 Hz, 4H), 1.70–1.63 (m, 4H), 1.41–1.27 (m, 20H), 0.88 (t, *J* = 7.0 Hz, 6H).

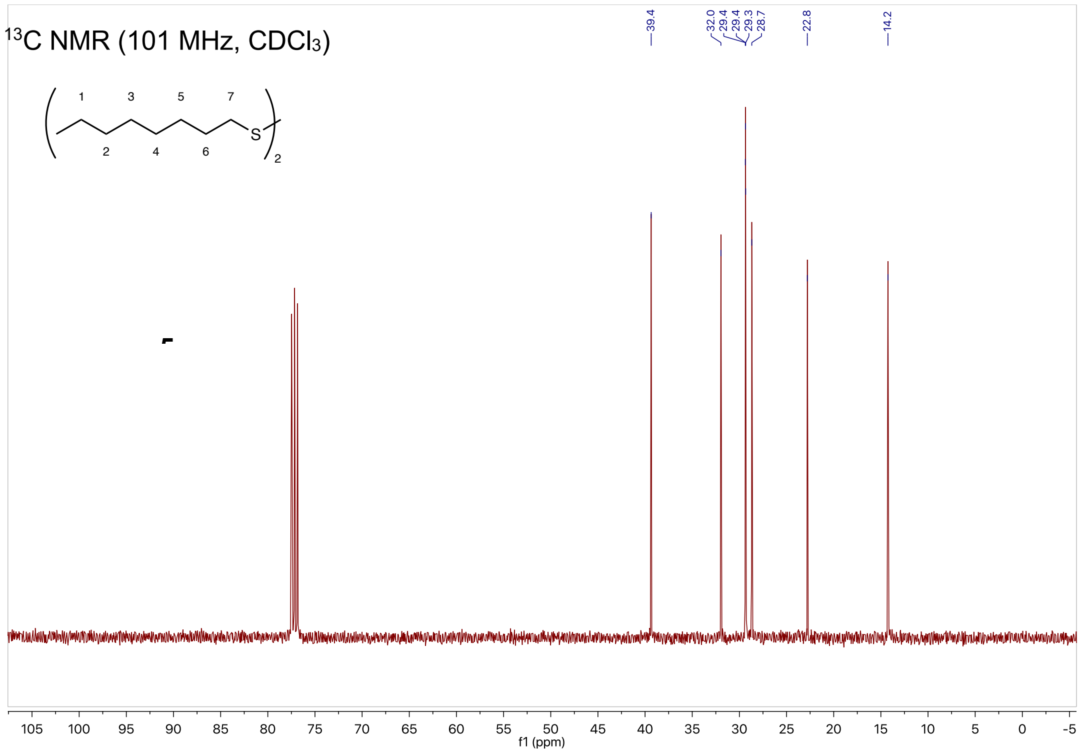

^{13}C NMR (101 MHz, $CDCl_3$): δ 39.4, 32.0, 29.4, 29.4, 29.3, 28.7, 22.8, 14.2.
